# Nicotine Pretreatment Increases Dysphoric Effects of Alcohol in Luteal-Phase Female Volunteers

**DOI:** 10.3390/ijerph6020526

**Published:** 2009-02-05

**Authors:** David M. Penetar, Elena M. Kouri, Elissa M. McCarthy, Michelle M. Lilly, Erica N. Peters, Trisha M. Juliano, Scott E. Lukas

**Affiliations:** Behavioral Psychopharmacology Research Laboratory, McLean Hospital/Harvard Medical School, 115 Mill Street, Belmont, MA 02478, USA; E-Mails: Kouri@hcp.med.harvard.edu (E.M.K.); E.McCarthy@va.gov (E.M.M.); grossmm@umich.edu (M.M.L.); enpeters@uvm.edu (E.N.P.); trisha.juliano@jefferson.edu (T.M.J.); lukas@mclean.harvard.edu (S.E.L.)

**Keywords:** Transdermal nicotine, alcohol, subjective effects, menstrual cycle, progesterone

## Abstract

The present report shows that nicotine enhances some of alcohol’s positive and negative effects in women and that these effects are most pronounced during the luteal phase of the menstrual cycle. Ten low progesterone and 10 high progesterone/luteal-phase women received nicotine patch pretreatments (placebo or 21 mg) 3 hours before an alcohol challenge (0.4 g/kg). Subjective effects were recorded on mood adjective scales and the Addiction Research Center Inventory (ARCI). Heart rate and skin temperature were recorded. Luteal-phase women reported peak positive (e.g. “stimulated”) and peak negative effects (e.g. “clumsy”, “dizzy”) almost twice as great as low progesterone women.

## 1. Introduction

The concurrent use of tobacco with alcohol is one of the most common drug combinations in the United States. There is a general consensus that nicotine modifies the acute effects of alcohol. This relationship is likely to be state- and dose-related as nicotine administration has been shown to result in both antagonism and synergism of alcohol’s effects [1–3]. Human laboratory studies designed to explore the nature of this interaction have frequently focused on how alcohol affects tobacco smoking or nicotine’s effects [3–7]. Mitchell *et al.* [8] demonstrated that alcohol consumption increases the number of cigarettes smoked in a controlled laboratory setting, but only shortly after drinking when blood alcohol levels were rising. Although Zacny *et al.* [9] demonstrated that alcohol did not increase tobacco cigarette preference over a money reinforcer, Burton and Tiffany [4] did show that alcohol intoxication increased cravings to smoke. The reverse situation of how nicotine affects alcohol intake has also been studied, but to a lesser extent [10, 11].

Acute administration of nicotine has been shown to potentiate the discriminative stimulus properties of ethanol in rats [12] and continuous administration of nicotine increases ethanol drinking by rats [13, 14]. With human subjects, Perkins *et al.* [3] showed that nicotine delivered intranasally increased alcohol’s subjective stimulating and cardiovascular effects. The subjective effects were noted when blood alcohol levels were rising shortly after drinking. Once drinking began, nicotine was found to reverse the sedating effects of alcohol during decreasing blood alcohol levels. The reversal of alcohol’s sedating effects was particularly prominent in men while the increased stimulating effects were more prominent in women (see also review in [15]). Kouri *et al.* [16] found that nicotine pretreatment via the transdermal patch enhanced both the subjective and cardiovascular effects of ethanol in men. Reports of feeling drunk and ethanol-induced euphoria, among other measures, were more prominent when subjects were pretreated with a 21 mg transdermal nicotine patch compared to placebo. Heart rate increases following drinking also were greater with nicotine pretreatment. The finding that tobacco smoking and alcohol consumption are positively correlated [17–20] has important health-related consequences, not only from the perspective of combined morbidity after chronic use and abuse, but may be important mediating factors in the initiation of using other drugs, particularly in adolescents.

Important sex-related differences in the effects of nicotine have been noted. In general, nicotine appears to be less of a reinforcer for maintaining cigarette smoking in women than in men [21] and this may be due to sex differences in the sensitivity to nicotine’s interoceptive cues [22]. Menstrual cycle hormones are important factors for understanding many drug effects in women. For example, responses to cocaine and amphetamine are influenced by varying estrogen and progesterone levels associated with the follicular and luteal phases [23–26]. Subjective responses to these drugs generally were reduced during the luteal phase of the cycle. In addition, drug intake has been shown to vary throughout the menstrual cycle. Both alcohol and nicotine use has been found to be greater during the luteal phase of the cycle [27–29], although this may not be a consistent effect with nicotine [30, 31]. Mello *et al.* [32], while examining cigarette smoking and alcohol self-administration in women, found that nearly three-quarters of women increased smoking during the late luteal phase of their menstrual cycle, as measured by inter-cigarette interval. Additional evidence for the influence of menstrual cycle phase on nicotine’s effects is seen during withdrawal. Perkins *et al.* [33] report that the severity of nicotine withdrawal symptoms is greater during the luteal (premenstrual) phase of the cycle than during the follicular, which may explain why others have reported more smoking during this phase [27, 34].

Nicotine administered by cigarette smoking has a relatively short duration of action. Peak blood nicotine levels are achieved typically within the time it takes to consume the cigarette (5 to 10 minutes) and decline quickly [35]. Alcohol, however, via the oral route, has a slower onset and longer duration of action. Therefore, in the present study, transdermal nicotine was used for several reasons. First, in an effort to study nicotine-alcohol interactions over a more sustained period of time, a steady-state of nicotine levels (as can be supplied by the patch) was desired for the duration of the assessment period. Second, cigarette smoking involves a host of other factors (sensations of taste, olfaction, intake of other chemicals, and strongly conditioned behaviors) that may interact with alcohol and the behavior of drinking, and thus affect subjective experiences. Third, with the availability of the nicotine patch over-the-counter, and the health and societal emphasis to restrict smoking, there is great likelihood that nicotine in forms other than smoked cigarettes (patch, smokeless tobacco, gum, and lozenge) will be used in conjunction with common drugs of abuse. Previous reports from our laboratory have shown that nicotine can enhance the subjective and physiological effects of marihuana in both men and women [36], of ethanol in men [16], and can attenuate the effects of cocaine [37]. The goals of the present study were to explore the effects of nicotine directly, unencumbered by the complexities of cigarette smoking, on alcohol’s subjective and physiological effects in women, and to examine the influences of progesterone on these effects. Our hypotheses were that nicotine pretreatment would alter alcohol’s effects and these effects would be further modulated by progesterone levels in women.

## 2. Experimental Section

*Participants*. Female participants were recruited from the Boston metropolitan area via newspapers, flyers and Internet advertisements. To be considered for the study, subjects had to be between the ages of 21 and 35 years old with a body mass index (BMI) between 18 and 25, report during an initial telephone interview regular smoking of at least five cigarettes per day and drinking at least four alcoholic drinks per week. Following the telephone interview, qualifying potential subjects were scheduled for a physical examination and a psychological evaluation in the laboratory. To qualify, subjects had to have a normal physical examination and electrocardiogram, have normal blood chemistry and urinalysis, have no current alcohol or other drug dependence (except nicotine dependence) according to DSM-IV criteria, have no major psychiatric disorder according to DSM-IV criteria (including depression), and report a negative maternal and paternal history of alcohol dependence. Subjects who met DSM-IV criteria for current or past alcohol abuse (but not dependence) were accepted. Subjects were excluded if they met DSM-IV criteria for premenstrual dysphoric disorder (PMDD), had a history of major head trauma, were hepatitis positive, or were regularly taking prescription medications (except oral birth control pills). Those women taking oral contraceptive medication (see below) had to be taking a combination type pill (estrogen plus progesterone); progesterone only pill-taking women were excluded. Information about those completing the study is presented in Table 1. For the duration of the study, all women kept track of their menstrual cycle with a daily menstrual calendar that included the start and stop dates of menses. The protocol and informed consent were reviewed and approved by the McLean Hospital Institutional Review Board. Participants were paid for their participation.

*Experimental design*. This study was a 3-visit, randomized dosing study investigating the effects of a 21 mg nicotine patch pretreatment versus a placebo patch on the subjective and physiological effects of an alcohol challenge (0.4 g/kg). The three conditions presented in a randomized order were: nicotine alone (21 mg nicotine patch and placebo drink), alcohol alone (placebo patch and 0.4 g/kg alcoholic drink), and a combination of both drugs (21 mg nicotine patch and 0.4 g/kg alcoholic drink). Two groups were studied: 10 women taking oral contraceptives who had progesterone levels of less than 1.9 ng/mL and 10 women not taking oral contraceptives who were in the luteal phase of their menstrual cycle. A blood sample was taken on every study day for serum progesterone levels to verify menstrual cycle phase (Quest Diagnostics, Cambridge, MA). For the low progesterone level women, study days were scheduled between days five and 10 of their cycle (Day 1 was the first day of menstruation). Their average progesterone levels on study days were 0.65±0.32 ng/mL (range: <0.3 – 1.9 ng/mL) (Table 1). Luteal participants used non-hormonal methods of contraception and kept track of their cycles through the use of a commercial ovulation kit (Answer One-Step Ovulation Test Kit). The study was scheduled to occur seven (7) to 13 days after ovulation (between days 21 and 27 of the menstrual cycle). Their average progesterone levels on study days were 7.14±4.33 ng/mL (range: 0.8 – 16 ng/mL). For each participant, study days occurred on or about the same day of their monthly menstrual cycle and thus took at least 3 months to complete all drug conditions.

*Study Day*. Participants were required to abstain from using all illicit drugs and alcohol for 72 and 24 hours, respectively, prior to the study. Participants could not smoke cigarettes, have any caffeine, eat or drink liquids (excluding water) from midnight prior to each study day. If they failed to comply with these restrictions, the study day was rescheduled. Participants arrived at the laboratory at 8:00 a.m. at which time they provided breath samples to check for the presence of alcohol (AlcoSensor, Intoximeter, Saint Louis, MO) and carbon monoxide as a measure of whether they complied with the smoking restrictions (Vitalograph, Lenexa, KS). Breath alcohol levels (BAL) had to be below 0.002% and expired air had to be below 14 ppm carbon monoxide for subjects to participate that day. A urine sample had to be negative for illicit drugs (Biosite Diagnostics, San Diego, CA) and pregnancy (QuPID, Stanbio Laboratory, Boerne, TX). All subjects met these study restrictions at every visit and no session had to be rescheduled due to failure to meet these study restrictions.

*Nicotine pretreatment*. Immediately following these evaluations, participants were taken into a dormitory-like room (equipped with a recliner chair, television, and stereo), fitted with skin temperature and heart rate devices for continuous monitoring (Mini Logger^®^ Series 2000; Mini Mitter Co., Bend, OR) and given a series of paper and pencil questionnaires to assess mood. Mood was assessed through the use of the Addiction Research Center Inventory (ARCI) [38] and visual analog scale (VAS) questionnaires. A research assistant then applied either a 21 mg nicotine (NicoDerm^®^ CQ, SmithKline Beecham) or placebo patch to the back of their upper arm. Placebo patches consisted of a nicotine patch without the plastic backing removed. All identifying markings were masked before a patch was used. Participants were provided a light breakfast (toast and juice) and for the first 2.5 hours of the study, participants were able to read, watch TV, or listen to music while relaxing in a recliner chair. At 30 minute intervals, the participants completed the ARCI and VAS questionnaires, and their blood pressure was taken. After 2.5 hours of nicotine pretreatment, participants were taken into a sound and light attenuated room equipped with a wired intercom and a closed circuit camera that provided auditory and visual contact with the participants. Each participant relaxed in a recliner chair where they were connected to an electrocardiogram machine, blood pressure cuff, and a skin temperature thermister. A catheter was inserted into an antecubital vein for blood collection using an integrative sampling procedure [39].

*Acute alcohol challenge*. Exactly three hours after the application of the nicotine or placebo patch, participants drank either an alcoholic drink or a placebo drink. The 400 mL alcoholic drink consisted of chilled orange juice mixed with a name brand vodka (80 proof) to a dose of 0.4 g/kg which was divided into 3 cups. (The drink is comparable to approximately two typical alcoholic drinks). The placebo drink consisted of three cups filled with an equivalent amount of juice. A small amount of vodka (0.25 mL) was placed on the surface of the juice and the rims of the glasses were swabbed with vodka to mask the taste of the placebo drink. When instructed, participants began drinking and were given five minutes to finish each cup, for a total of a 15-minute alcohol administration period. Fifteen minutes after alcohol administration was complete (30 minutes post-onset of drinking), blood pressure was taken, participants were asked to answer a set of questionnaires using a keypad, and then were asked to give a breath sample to measure alcohol concentration levels. Every thirty minutes thereafter, participants had their blood pressure taken, were asked to answer questionnaires (ARCI, VAS) and give a breath sample. Three hours after the beginning of the drinking session, the participants were told the study was over, were given lunch, and then sent home in a cab. Breath alcohol concentration levels had to be below 20 mg/dL (0.02%) before the participants were allowed to leave the laboratory. All participants were transported to and from the laboratory by taxicab.

*Subjective measures*. On 12 separate occasions during each study day (every 30 minutes from baseline through the end of the session), participants were administered the Addiction Research Center Inventory (ARCI) and a set of Visual Analog Scales (VAS). The 49-item ARCI questionnaires were scored to give ratings on MBG (morphine/benzedrine group-euphoria), PCAG (pentobarbital/chlorpromazine/alcohol group--sedation), AMP (amphetamine--stimulation), BG (benzedrine group--stimulation), and LSD (lysergic acid diethylamide--dysphoria and somatic effects) scales. Participants rated their response to the following 20 adjectives/phrases on a 100-point VAS from “not at all” to “extremely,”: happy, stimulated, anxious, clumsy, dizzy, drunk, great, high, muddled/confused, nauseous, terrible, sleepy, floating, slurred speech, uncomfortable, feel effects of alcohol, desire to use alcohol, desire not to use alcohol, desire to smoke a tobacco cigarette, and desire not to smoke a tobacco cigarette.

Participants were also asked to report alcohol’s subjective effects on a continuous basis using a keypad device. Participants were instructed to press the key on the keypad labeled “euphoric” whenever they experienced feelings of intense well-being, good effects, or intense pleasure, and to press a key labeled “dysphoric” if they experienced intense bad feelings, bad effects, or discomfort. Participants were instructed to press the key labeled “detect” if at any point after the onset of the drinking session they felt the effects of alcohol, and to turn it off when they no longer felt the effects of alcohol. A computer screen in the experimental chamber provided participants with a record of their keypad report, and all three could be registered at the same time, if appropriate.

*Physiologic measures*. Skin temperature (°C) and heart rate (beats per minute or bpm) were recorded throughout the study using a Mini Logger^®^ Series 2000 device (Mini Mitter Co., Bend, OR). Skin temperature was recorded using single-use temperature probes (Steri-Probe, Cincinnati Sub-Zero Products, Cincinnati, OH) attached to the stomach area just below the lowest right rib and connected to a port in the logger. Heart rate was recorded via a Polar Co. chest belt sensor attached (via Cleartrace adult ECG electrodes; ConMed Corp., Utica, NY) to the left side of the participant’s chest below the heart. While in the chamber, participants also had a three-lead EKG (Lead III), and a blood pressure cuff attached to their right arm. Blood pressure was taken at 30-minute intervals either manually during the nicotine pretreatment period or with an automated sphygmomanometer (Hewett Packard 78352A physiological recording device) during the alcohol challenge period.

*Blood sampling procedures/plasma analysis*. An intravenous (i.v.) catheter (Kowarski-Dakmed Thromboresistant Blood Withdrawal Needle and 8 foot Tubing Set; Dakmed, Inc., Buffalo, NY) was inserted in an antecubital vein for withdrawal of blood samples after 2.5 hours of patch pretreatment and once the participant was taken into the experimental chamber. The distal end of the catheter was passed through an opening in the chamber wall and attached to a 10-mL syringe mounted on a withdrawal syringe pump at a rate of 1 mL/min. Syringes were changed every five minutes and the blood was put into Vacutainer^®^ tubes containing sodium fluoride and then placed on ice (see [39]). The blood samples were then centrifuged and the plasma was separated into a plastic vial and sent to Quest Diagnostics (Cambridge, MA) for quantitative ethanol analysis through gas chromatography. The sensitivity of the assay was 10 mg/dL with an intersample variation of 4.1%. Selected blood samples were analyzed also for quantification of nicotine levels through gas chromatography by National Medical Services (Willow Grove, PA). The sensitivity of the assay was 5 ng/mL with an intrasample variation of 6.3% and an intersample variation of 9.7%. Blood samples used to verify progesterone levels were taken at the beginning of the study and were analyzed by Quest Diagnostics (Cambridge, MA).

*Data analysis*. Dependent variables were analyzed using a repeated measures ANOVA (SPSS 11.0 for Mac OS X). Dose and time were treated as within-subject factors and progesterone level (low vs. high) was treated as a between subjects factor. In order to facilitate and simplify analysis of the time course effects for the physiological data, separate analyses were performed on the nicotine pretreatment (before the drinking period) time points and then on the post-alcohol drinking time points. The post-drinking analysis included the baseline (–200 minute data). Analysis of the peak subjective and physiologic responses was performed using a repeated measures ANOVA. Again, dose and time were treated as a within-subjects factor and progesterone level was treated as a between subjects factor. Following the identification of a main effect, Fisher’s Least Significant Differences post-hoc tests were performed to identify where significant time point or dose effects occurred. Statistical significance was set at p≤0.05.

## 3. Results

### 3.1. Effects following pretreatment with nicotine patch

Nicotine was without effect on the subjective measures but did produce a significant elevation in heart rate (statistical analysis results for the physiological measures are reported in Table 2). There were no group differences at baseline (before nicotine patch application). Shortly after the nicotine patch application (within 20 minutes), heart rates increased significantly over baseline conditions and remained elevated for the duration of the pretreatment period. Average increases ranged from 9–15 beats per minute (bpm). Low progesterone women had higher heart rates following nicotine patch administration than high progesterone/luteal-phase women with average differences between 4 and 12 bpm. This heart rate increase was accompanied by a significant increase in systolic blood pressure following nicotine administration. Differences between the no nicotine and nicotine pretreatment conditions were small but consistent: between 4 and 6 mm Hg. Diastolic blood pressure was not affected by nicotine pretreatment. There were no differences in blood pressure due to progesterone levels/menstrual cycle phase.

### 3.2. Alcohol and combined nicotine-alcohol effects: Subjective measures

Visual analog scales. Except for ratings on the ‘desire to smoke’ scale, nicotine did not produce any significant changes on the visual analog scales during pretreatment; ratings on the scale adjectives remained stable during the pretreatment period regardless of whether participants received nicotine or not. Ratings on the ‘desire to smoke’ scale gradually decreased from baseline (when the participants first arrived in the laboratory) and were significantly lower at the last assessment before drinking in both groups of women and for both the placebo and active nicotine pretreatment conditions [time: F (5, 90) = 16.621, p<.001].

**Figure 1.**
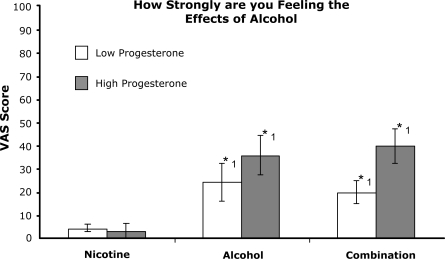

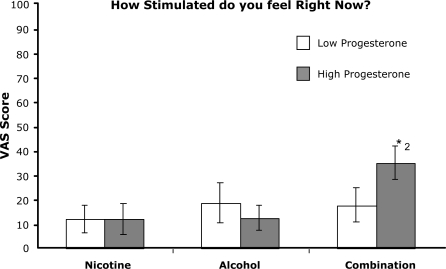

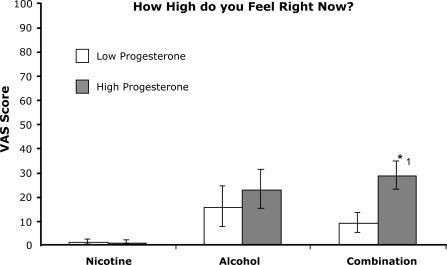

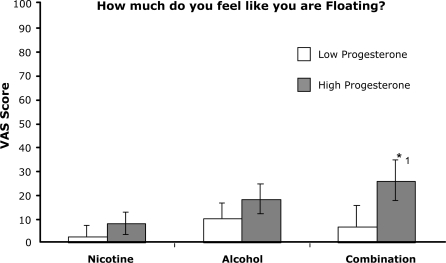
Peak effects scores for 4 scales of euphoric effects: A-Feeling Alcohol Effects; B-Stimulated; C-High; D-Floating. Participants rated their subjective effects on a 100 point scale from 0 – “not at all” to 100 – “extremely”. Columns are averages (± sem) by drug condition and progesterone group. *1: Drug condition significantly different from nicotine alone (p<.05). *2: Combination drug condition significantly different from nicotine alone and alcohol alone (p<.05). a. Self-reported feelings of ‘alcohol effects’. b. Self-reported feelings of ‘Stimulated’. c. Self-reported feelings of ‘High’. d. Self-reported feelings of ‘Floating’.

An analysis of the peak effects following the alcohol drink revealed significant dose effects for 4 scales assessing euphoric conditions (Figure 1). Responses following the nicotine-alcohol combination were significantly greater than the nicotine alone condition for ‘feel the effects of alcohol’ [F (2, 36) = 13.179, p<.001], ‘stimulated’ [F (2, 36) = 3.831, p=.031], ‘high’ [F (2, 36) = 7.788, p=.002], and ‘floating’ [F (2, 36) = 3.344, p=.047]. Additionally, responses on ‘feel effects of alcohol’ for the alcohol alone condition were significantly greater than the nicotine alone condition. Responses to ‘drunk’ were similar to ‘feel effects of alcohol’ and were significantly higher in both alcohol conditions in comparison to the nicotine alone condition [F (2, 36) = 8.986, p=.001] (data not shown). Although responses from the participants with low progesterone levels were generally lower than the high progesterone participants, there were no significant differences between the two groups on these measures of subjective alcohol effects. Ratings of ‘desire to smoke a tobacco cigarette’ were significantly higher in the alcohol alone condition in comparison to either the nicotine alone or the combination drug condition [F (2, 36) = 6.705, p =.003] (data not shown). Progesterone did not alter this effect.

**Figure 2.**
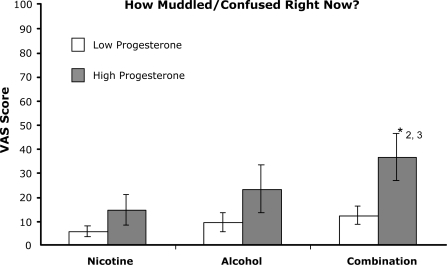

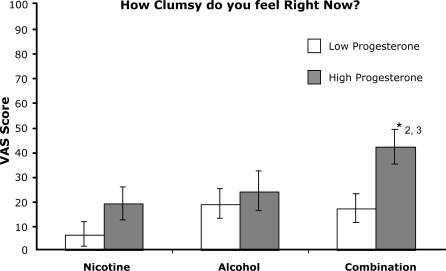

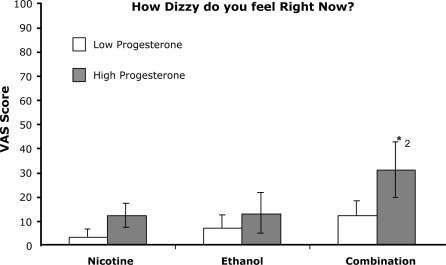

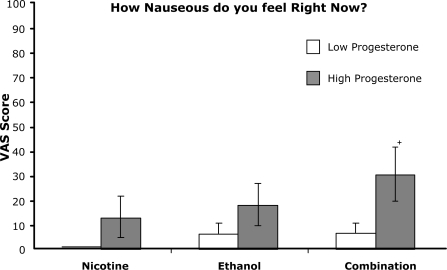
Peak effect scores for 4 scales of dysphoric effects: A-Muddled/Confused; B-Clumsy; C-Dizzy; D-Nauseous. Participants rated their subjective effects on a 100 point scale from 0 – “not at all” to 100 – “extremely”. a. Self-reported feelings of ‘Muddled/Confused’. b. Self-reported feelings of ‘Clumsy’. c. Self-reported feelings of ‘Dizzy’. d. Self-reported feelings of ‘Nauseous’. Columns are averages (± sem) by drug condition and progesterone group. *2: Combination drug condition significantly different from nicotine alone and alcohol alone (p<.05). *3: Significant difference between low and high progesterone groups (p<.05). +Statistical trend for combined dose different from nicotine alone condition (p=.062) and for difference between progesterone groups (p=.068).

Three of the scales assessing dysphoric effects showed significant dose effects for the combination drug condition (Figure 2). ‘Muddled/confused’ [F (2, 36) = 3.628, p=.037], ‘clumsy’ [F (2, 36) = 4.568, p=.017], and ‘dizzy’ [F (2, 36) = 4.252, p=.022] ratings were significantly higher following the combination drug condition than either the nicotine or alcohol alone conditions. Ratings of ‘nauseous’ showed a trend toward higher ratings in the combination condition in comparison to the nicotine alone condition [F (2, 36) = 3.0, p=.062]. In addition, ratings on ‘muddled/confused’ and ‘clumsy’ were significantly higher in the high progesterone group in comparison to the low progesterone group in the drug combination condition.

*Keypad*. Self-reported detection of an alcohol effect and the duration of that effect were dependent on the administration of alcohol. In the nicotine alone condition (placebo drink), three low progesterone women and two high progesterone women reported feeling an alcohol drug effect with latency to detection averages of 130.1 ± 75.1 and 165.2 ± 43.3 minutes respectively. The drinking session (placebo) occurred at time 30. Following alcohol drinking, participants reported detecting an alcohol drug effect significantly faster [F(2, 34)=30.853, p<.001] with 8 of 10 low- progesterone and 10 out of 10 high progesterone women detecting an alcohol effect in this condition. Latency to detection times were 57.9 ± 69.5 and 20.5 ± 14.8 minutes respectively. This effect was not altered by nicotine pretreatment. In the combined drug condition, 8 out of 9 low progesterone women and 9 out of 10 high progesterone women reported detecting an alcohol drug effect with average times of 43.8 ± 54 and 34.1 ± 52.5 minutes respectively. (Data from one low progesterone participant was lost due to equipment problems). Duration of an alcohol drug effect for low- and high progesterone women was 64.2 ± 50.7 and 80.9 ± 56.2 minutes in the alcohol-alone condition, and 47.9 ± 47.8 and 69.4 ± 52.7 minutes in the combined nicotine-alcohol condition. Both alcohol conditions were significantly longer than the nicotine-alone condition [F(2, 34)=10.942, p<.001]. Progesterone levels did not affect detection latencies or duration of effect. Reports of euphoria and dysphoria were low in all conditions (between 20 and 30%). Time to onset of these conditions and duration of these conditions when reported were not altered by drug condition and were not different between the two progesterone groups.

*ARCI*. Scores for the PCAG (pentobarbital/chlorpromazine/alcohol group--sedation), AMP (amphetamine--stimulation), BG (benzedrine group--stimulation), and LSD (lysergic acid diethylamide--dysphoria and somatic effects) scales were unaffected by drug administration (data not shown). For the MBG scale, scores gradually decreased over the course of the experiment from a high at baseline of 2.5 to 1.7 near the end of the session (data not shown). A slight, but non-significant increase in MBG scores was observed immediately following alcohol administration but was not dose related [dose: F(2, 36)=.709, p=.499; time: F(11, 198)=1.986, p=.032] Overall, high progesterone women reported MBG scores 2 to 3 times higher than that of low progesterone women [F(1, 18)=4.889, p=.040].

### 3.3. Alcohol and combined nicotine-alcohol effects: Physiologic variables

**Figure 3.**
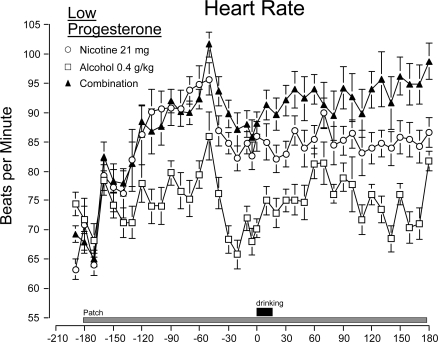

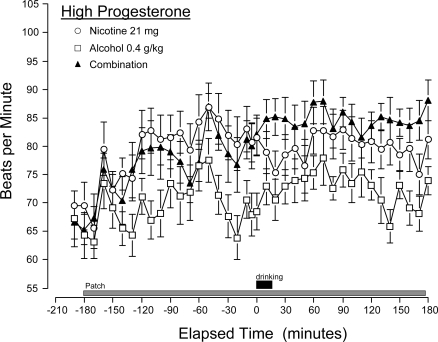
Time course of heart rate by drug condition (means ± sem) and progesterone levels: A-Low Progesterone; B-High Progesterone/Luteal-Phase women. Heart rates at baseline (–200 minutes) are not significantly different between groups. The nicotine patch significantly increased heart rates during the pre-drinking time period (p<.001). For both groups after the drinking period, the three drug conditions are significantly different from each other (p<.001). The large increase in heart rates at −50 minutes was due to a bathroom break given to all subjects. a. Heart rate responses in Low Progesterone women to three drug conditions. b. Heart rate responses in High Progesterone/Luteal-Phase women to three drug conditions.

*Heart rate*. Nicotine pretreatment significantly increased heart rate. In addition, significant increases in heart rate above baseline were observed in all three conditions following drinking (Figure 3, Table 2). The three dose conditions were significantly different from each other. Increases were greatest for the combined nicotine patch-alcohol condition, intermediate for the nicotine alone condition and least for the alcohol alone condition. A significant 3-way interaction (dose x time x progesterone level) was observed following the drinking procedure indicating the greater heart rate changes in the low progesterone women. Analysis of the peak heart rates following drinking revealed that heart rates in comparison to baseline increased on the average 14.9 bpm for the alcohol alone condition, 21.2 bpm for the nicotine alone condition, and 26.8 bpm for the combination drug condition representing increases of 23%, 31% and 39% respectively. Peak rates were observed on individual participants between 30 and 90 minutes after drinking.

*Blood pressure*. Both systolic and diastolic blood pressure were affected similarly: the nicotine alone and the nicotine plus alcohol conditions were significantly higher than the alcohol alone condition (Table 2). During these conditions, systolic pressure was significantly elevated over baseline for one hour. Differences were between 7 and 12 mm Hg. Diastolic pressure was not significantly elevated over baseline, but the 30 minute values were higher by between 2 and 3 mm Hg than values subsequent to 90 minutes after administration. There were no differences due to progesterone levels.

*Skin Temperature*. Overall, skin temperature rose during the pretreatment phase, from the beginning of the study with an average of 33.8° C to an average of 35.5° C at 60 minutes before drinking (Table 2). Temperatures of participants receiving the active nicotine patch showed significantly less of an increase compared to those receiving the placebo patch, although the differences were modest (in the range of a half degree Celsius). Following alcohol drinking, temperatures generally remained stable, although the temperature of the subjects drinking alcohol decreased slightly in comparison to the nicotine alone condition at 80 minutes after ingestion and remained depressed for 30 minutes. Differences again were approximately a half degree Celsius. There were no differences in skin temperature due to progesterone levels.

### 3.4. Blood analysis

Blood samples were analyzed for nicotine concentrations before application of the patch to verify overnight abstinence and after application of the patch. Nicotine was not detected in any participant, except one, at the start of the study day in agreement with refraining from smoking for at least 8 hours. The participant who tested positive had a level of 5.6 ng/mL at the start of the session (which is less than the 7 ng/mL level set by Marks *et al.* [40] as evidence of overnight abstinence). Her pretreatment data were inspected and found not to differ from other participants’ results. When participants received the active patch, nicotine plasma levels averaged between 23 and 25.5 ng/mL at 2.5 hours after application and rose slightly to averages between 25.25 and 31.9 ng/mL at the end of the session (6 hours after application). There were no significant differences in nicotine levels between nicotine alone and nicotine plus alcohol drinking sessions, nor were there any differences based on progesterone levels/menstrual cycle phase.

Blood alcohol levels (BAL) rose quickly after drinking, reaching peak concentrations at 50 minutes after commencement. Peak concentrations for the alcohol groups averaged between 45 and 55 mg/dL (equivalent to 0.045 and 0.055%). Neither nicotine pretreatment nor progesterone levels altered the BAL concentration curves. Other standard pharmacokinetic parameters were analyzed: time to maximum concentration, elimination half life, and area under the plasma concentration curve. None of these measures was significantly altered by nicotine pretreatment and there were no differences due to progesterone levels.

## 4. Discussion

This study shows that alcohol drinking produces significant changes in subjective and physiological measures in women, and that some of these effects are increased by nicotine pretreatment. Furthermore, this study shows that progesterone levels can alter the magnitude of these responses with effects greatest for high progesterone/luteal-phase women.

In the previous study from our laboratory [16], men were studied in the identical paradigm using the same nicotine pretreatment patch (21 mg) but including two doses of alcohol (0.4 and 0.7 g/kg). In that study, nicotine pretreatment enhanced alcohol effects after both doses. The present study used only the lower dose of alcohol and used two groups of female participants to study the influence of progesterone levels. A comparison of the lower alcohol dose effects between men in the previous study and the two groups of women in the present study shows many similarities. Increased reports of feelings of ‘drunk’, ‘feeling the effects of alcohol’, and ‘desiring to drink alcohol’ after drinking were observed after the nicotine condition in both the women in the present study and the men in our previous study. However, in contrast to the men in the previous study, the women in this study reported an increased feeling of ‘high’, ‘stimulated’, and ‘floating’ after consuming alcohol when pretreated with nicotine. Also, in contrast to the men in the previous study, women reported more instances of negative effects. Significant increases of ‘muddled/confused’, ‘clumsy’, and ‘dizzy’ were also observed. These effects were more predominant in the group of high progesterone women. Comparisons of peak subjective reports after the nicotine-alcohol condition show that responses from the high progesterone/luteal-phase women are similar to those from the men. In contrast, peak responses from the women in the low progesterone group showed the least amount of change, roughly half the magnitude of the responses from the men and the high progesterone women. Secondly, women overall were more sensitive to the negative effects of nicotine-alcohol combinations than men, and these effects are significantly greater in high progesterone/luteal-phase women.

Studies with human volunteers also have shown that there exists a complex interaction between nicotine and alcohol. Perkins *et al*. [3] demonstrated that a combination of nicotine via nasal spray plus alcohol had differential effects in men vs. women. Nicotine reversed the decreases in vigor and arousal that alcohol produces in men, whereas in women alcohol alone was not found to decrease vigor and arousal, and nicotine significantly increased these effects. Using the nicotine patch which delivers nicotine at a slow and sustained rate, Kouri *et al*. [16] reported that nicotine enhanced several of alcohol’s effects in men. Subjective effects of feeling drunk, feeling the effects of alcohol, and desire to drink more alcohol were increased with nicotine pretreatment. The present study shows that slow delivery of nicotine in abstinent smokers did not produce significant subjective effects by itself, but when combined with alcohol, there tended to be an increase in alcohol’s typical responses. The present study furthermore shows that these increases are particularly prominent in high progesterone/luteal-phase women.

Nicotine and alcohol had additive effects on heart rate but not on blood pressure. Blood pressure changes were due to nicotine administration and it was unaltered by the addition of alcohol at the dose employed in this study. Previous studies have also studied the effects of these drugs on heart rate and blood pressure. Benowitz *et al.* [41], using higher doses of alcohol (0.5 and 1.0 g/kg), found that alcohol alone increased heart rate and systolic blood pressure and that alcohol plus intravenous nicotine together produced an additive effect. Perkins *et al.* [3], also using a slightly higher dose (0.5 g/kg), found an alcohol-nicotine (via nasal spray) additive effect on heart rate but not on blood pressure as increases from baseline under the combined condition were similar to those following nicotine alone. Additionally, Perkins *et al.* [3] did not find any differences between men and women. Our studies with male and female subjects are consistent with these studies and provide further evidence for a heart rate increase by nicotine-alcohol combinations in comparison to either drug alone.

The direct effects of progesterone on nicotine and/or alcohol’s effects have not been extensively studied. Nicotine (via the patch) has been shown to be more effective in relieving symptoms during a 5-day abstinence period when progesterone levels are high (late luteal phase of the menstrual cycle) [42]. Sofuoglu *et al*. [43] found that progesterone treatments during the follicular phase (when progesterone levels are low) reduce cravings and other subjective effects of smoking. The present study suggests that progesterone influences responses to nicotine-alcohol combinations. High progesterone women showed a greater peak response to both positive and negative subjective effects such as feelings of floating, stimulation, confused, and clumsy than the low progesterone women. Additionally, high progesterone women reported detecting alcohol effects sooner and for a longer period of time although this was not significantly different from the low progesterone women. Several studies using nicotine or alcohol alone stand in contrast to the present findings in that subjective and physiological responses were not found to differ as a function of menstrual cycle phase. Marks *et al.* [40] found that subjective and physiological responses to nicotine nasal spray were not affected by menstrual cycle phase. Analysis of responses on visual analog scale adjectives, and physiological measures of heart rate and blood pressure were similar across early follicular, mid-follicular, mid-luteal, and late luteal phases. An analysis of alcohol’s effects during these same four phases of the menstrual cycle also showed no differences on subjective measures (e.g. ARCI, Profile of Mood States) and physiological variables (e.g. heart rate, blood pressure) [44]. Other behaviors such as flight simulator performance and attention do not change during the menstrual cycle [45–47]. Studies of a menstrual cycle phase analysis of nicotine-alcohol combinations are lacking and the present study suggests that there are effects not evident when either drug is studied alone.

An exploration of the effects of nicotine and alcohol combinations on subjective variables and physiological measures will help understand how these two commonly used drugs can influence each other, shedding light on why the intake of this combination is so often observed. Differences between men and women, and the influence of the menstrual cycle hormones in women are important considerations in developing a complete picture of drug intake and patterns. The data collected for low and high progesterone women indicate that responses to nicotine-alcohol combinations can be highly variable. It is interesting to note that in the present study high progesterone/luteal-phase women had higher subjective responses to the alcohol-nicotine combination (both positive and negative responses) compared to the low progesterone women in spite of lower magnitude increases in heart rate. Larger sample sizes may be required to fully assess the influence of menstrual cycle hormones and their interactions with subjective and physiological responses. Original sample sizes were computed based on reports at the time (e.g. [3]) and previous experience with these measures (e.g. [48]). Results from more recent studies of menstrual cycle phase differences (e.g. [33, 44]) and the current study would indicate that larger sample sizes are necessary to properly assess the influence of menstrual cycle hormones. Several other limitations of the study should be noted. A full within-subjects design where women were tested on all three drug combinations in each of two menstrual cycle phases (follicular and luteal) was not employed. Only one dose of alcohol was tested as was only one strength of the nicotine patch. Additionally, there was no double-placebo condition. These design restrictions were employed in an effort to complete the study in a timely manner and to reduce the demands on individual participants. The results should be viewed with these limitations understood and additional studies are necessary to verify the present findings.

Two other factors should be noted. First, although there was a clear and significant difference in progesterone levels between the two groups, the average level in the luteal-phase women of our study (7.14 ng/mL) was at the lower end of what has been typically reported (7.5 – 15 ng/mL) during the luteal phase of the menstrual cycle (e.g. [23, 42, 44, 49, 50]). Despite this, and the wide range of values in our subjects, significant differences emerged between the groups. A more complete assessment of nicotine-alcohol interactions may be obtained in future experiments when progesterone levels are increased and better controlled. Second, the influences of estrogen were not assessed in this study. Participants took several different brands of oral contraceptive medications and the amount of estrogen undoubtedly varied. Although the direct, isolated effects of estrogen/estradiol administration on responses to alcohol have not been studied in humans, most recent studies indicate that subjective responses to alcohol alone do not vary across the menstrual cycle (see review [51]). Our results are consistent with those reports. Subjective effects to alcohol alone were similar in both groups. Significant phase effects emerged only following nicotine pretreatment. The effects of estrogen alone on subjective responses, if any, are believed to be small, but await further experimentation.

## 5. Conclusions

The present study was accomplished as part of a set of experiments exploring the effects of nicotine pretreatment on the effects of several drugs of abuse [16, 36, 37]. They were designed to help understand why nicotine is so often used in conjunction with other drugs and have demonstrated that nicotine pretreatment does, in fact, alter alcohol’s subjective and physiological effects. Perkins [15] has reviewed data to indicate that nicotine increases subjective and physiological responses to alcohol. The present study agrees with these results and may contribute additional data on understanding how sex differences and menstrual cycle hormones in women are important factors to understanding the nature of the nicotine-alcohol interactions.

## Figures and Tables

**Table 1.** Demographic and Drug Use Information (averages ± sd). There were no significant differences between groups on any demographic or drug use variable. Caffeine use was defined as the number of 100 mg drinks per day. FTND: Fagerstrom Test for Nicotine Dependence.

	Age (years)	Cigarettes per Day	FTND Score	Caffeine Use (drinks/day)	Alcohol drinks per week	Met Criteria for Current or past Alcohol Abuse	Progesterone Levels on Study Days* (ng/mL)
Low Progesterone	22.3±1.5	15.3±4.7	4.2±1.1	1.90±0.94	8.8±5.8	3	M=0.65±0.32 MDN=0.6
High Progesterone (Luteal-phase women)	24.1±2.8	14.2±3.2	4.0±1.3	2.25±1.64	11.2±5.6	5	M=7.14±4.33 MDN=7.45

* Significantly different averages between groups, p<.001. M=mean (average) value; MDN=median value.

**Table 2.** Physiological Measures: Statistical Results. Separate analyses were performed on the time points before the drinking period, and on the time points after drinking. The after drinking analyses included the baseline time point before patch administration (–200 minute time point) and all after-drinking time points. Prog = progesterone factor.

Heart Rate	Before Drinking	Dose:	F(2, 36)=26.647, p<.001

		Time:	F(19, 342)=44.451, p<.001
		Dose x Time:	F(38, 684)=7.097, p<.001
		Dose x Time x Prog:	F(38, 684)=1.737, p=.004
	
	After Drinking	Dose:	F(2, 36)=45.619, p<.001
	
		Time:	F(19, 342)=18.917, p<.001
		Dose x Time:	F(38, 684)=3.924, p<.001
		Dose x Time x Prog:	F(38, 684)=1.489, p=.031

Systolic Blood Pressure	Before Drinking	Dose:	F(2, 36)=3.968, p=.028

		Time:	F(5, 90)=16.314, p<.001
		Dose x Time:	F(10, 180)=3.129, p=.001
		Dose x Time x Prog:	F(10, 180)=0.584, p=.826
	
	After Drinking	Dose:	F(2, 36)=5.593, p=.008
	
		Time:	F(5, 90)=7.048, p<.001
		Dose x Time:	F(10, 180)=1.493, p=.145
		Dose x Time x Prog:	F(10, 180)=1.178, p=.308

Diastolic Blood Pressure	Before Drinking	Dose:	F(2,36)=1.805, p=.179

		Time:	F(5,90)=2.132, p=.069
		Dose x Time:	F(10,180)=0.655, p=.765
		Dose x Time x Prog:	F(10,180)=0.934, p=.504
	
	After Drinking	Dose:	F(2, 36)=7.395, p=.002
	
		Time:	F(5, 90)=3.804, p=.004
		Dose x Time:	F(10, 180)=1.338, p=.069
		Dose x Time x Prog:	F(10, 180)=0.666, p=.755

Skin Temperature	Before Drinking	Dose:	F(2, 36)=5.034, p=.012
	
		Time:	F(18, 324)=48.240, p<.001
		Dose x Time:	F(36, 648)=0.800, p=.793
		Dose x Time x Prog:	F(36, 648)=1.224, p=.064
	
	After Drinking	Dose:	F(2, 36)=1.978, p=.153
	
		Time:	F(18, 324)=5.636, p<.001
		Dose x Time:	F(36, 648)=1.496, p=.033
		Dose x Time x Prog:	F(36, 648)=0.495, p=.995
